# Whole Genome Sequence Analysis of Salmonella Enteritidis PT4 Outbreaks from a National Reference Laboratory’s Viewpoint

**DOI:** 10.1371/currents.outbreaks.aa5372d90826e6cb0136ff66bb7a62fc

**Published:** 2015-09-11

**Authors:** Véronique Wuyts, Sarah Denayer, Nancy H.C. Roosens, Wesley Mattheus, Sophie Bertrand, Kathleen Marchal, Katelijne Dierick, Sigrid C.J. De Keersmaecker

**Affiliations:** Department of Microbial and Molecular Systems, Centre of Microbial and Plant Genetics, KU Leuven, Leuven, Belgium; Department of Plant Biotechnology and Bioinformatics, Ghent University, Ghent, Belgium; Platform Biotechnology and Molecular Biology, Scientific Institute of Public Health (WIV-ISP), Brussels, Belgium; Scientific Institute of Public Health (WIV-ISP), Scientific Service of Foodborne Pathogens, NRL Foodborne Outbreaks, Brussels, Belgium; Scientific Institute of Public Health (WIV-ISP), Platform Biotechnology and Molecular Biology, Brussels, Belgium; National Reference Centre for Salmonella and Shigella, Bacterial Diseases Division, Communicable and infectious Diseases, Scientific Institute of Public Health, Brussels, Belgium; National Reference Centre for Salmonella and Shigella, Bacterial Diseases Division, Communicable and Infectious Diseases, Scientific Institute of Public Health (WIV-ISP), Brussels, Belgium; Department of Plant Biotechnology and Bioinformatics, Ghent University, Ghent, Belgium; Department of Information Technology, Ghent University, IMinds, Ghent, Belgium; Scientific Institute of Public Health (WIV-ISP), Scientific Service of Foodborne Pathogens, NRL Foodborne Outbreaks, Brussels, Belgium; Scientific Institute of Public Health (WIV-ISP)

**Keywords:** disease outbreak, infectious disease, Salmonella, Whole genome sequence

## Abstract

Introduction: In April and May 2014, two suspected egg-related outbreaks of Salmonella enterica subsp. enterica serovar Enteritidis (S. Enteritidis) were investigated by the Belgian National Reference Laboratory of Foodborne Outbreaks. Both the suspected food and human isolates being available, and this for both outbreaks, made these the ideal case study for a retrospective whole genome sequencing (WGS) analysis with the goal to investigate the feasibility of this technology for outbreak investigation by a National Reference Laboratory or National Reference Centre without thorough bioinformatics expertise.

Methods: The two outbreaks were originally investigated epidemiologically with a standard questionnaire and with serotyping, phage typing, multiple-locus variable-number of tandem repeats analysis (MLVA) and antimicrobial susceptibility testing as classical microbiological methods. Retrospectively, WGS of six outbreak isolates was done on an Illumina HiSeq. Analysis of the WGS data was performed with currently available, user-friendly software and tools, namely CLC Genomics Workbench, the tools available on the server of the Center for Genomic Epidemiology and BLAST Ring Image Generator (BRIG).

Results: To all collected human and food outbreak isolates, classical microbiological investigation assigned phage type PT4 (variant phage type PT4a for one human isolate) and MLVA profile 3-10-5-4-1, both of which are common for human isolates in Belgium. The WGS analysis confirmed the link between food and human isolates for each of the outbreaks and clearly discriminated between the two outbreaks occurring in a same time period, thereby suggesting a non-common source of contamination. Also, an additional plasmid carrying an antibiotic resistance gene was discovered in the human isolate with the variant phage type PT4a.

Discussion: For the two investigated outbreaks occurring at geographically separated locations, the gold standard classical microbiological subtyping methods were not sufficiently discriminative to distinguish between or assign a common origin of contamination for the two outbreaks, while WGS analysis could do so. This case study demonstrated the added value of WGS for outbreak investigations by confirming and/or discriminating food and human isolates between and within outbreaks. It also proved the feasibility of WGS as complementary or even future replacing (sub)typing method for the average routine laboratory.

## Introduction

For 71% of the total number of outbreaks reported within the European Union the causative agent is known. The most frequently reported causative agent of foodborne outbreaks remains *Salmonella*, with *Salmonella enterica* subsp. *enterica* serovar Enteritidis (*S*. Enteritidis) as predominant serovar. Egg and egg-related products are still the most common source (60%) of *S*. Enteritidis outbreaks in Europe^1^; this despite the implementation of the national control programme on reduction of Salmonella in commercial laying hens, with obligatory vaccination for countries with high incidence of *Salmonella* in laying hen flocks since 2008.

During outbreak investigations, in addition to collecting epidemiological information through interrogation of the human cases, bacterial isolates collected from food samples, leftovers and human cases (often via stool samples) are being characterised by the National Reference Laboratories for Food (NRL) and the Human Reference Centres (NRC) in order to find a common source of contamination to be able to control the outbreak as soon as possible. This allows to support a strong relatedness between the isolate from the human case and that from the suspected food, which can have important economic implications. It will also allow to identify other human cases linked to the outbreak, *i* *.e.* which consumed the same contaminated food. This is especially important for outbreaks with a dispersed geographical distribution of human cases. Several methods, including molecular ones, can be used for characterisation, or subtyping, of a pathogenic isolate. For S. Enteritidis, this concerns for example phage typing, multi-locus sequence typing (MLST) and multiple-locus variable-number of tandem repeats analysis (MLVA)^2^^,^ 3 ^,^ 4. However, in addition to some other disadvantages^2^, the resolution of these methods is not always sufficient to discriminate the outbreak isolates from the circulating background strains, especially when it concerns isolates belonging to the most frequently occurring subtypes.

Recently, whole genome sequencing (WGS) has been postulated as the universal, ultimate resolution subtyping technique^4^^,^ 5 ^,^ 6. However, its data analysis requires appropriate tools, often involving the necessary bioinformatics expertise which is not always present in the average routine laboratory. In general, for bacterial WGS analysis, two workflows are proposed in literature^7^, *i.e.* allele-based (comparison of allelic variants) or single nucleotide polymorphism (SNP)-based (SNP calling). For an allele-based WGS analysis, which is also called gene-by-gene comparison or whole genome or core genome MLST (cgMLST), depending on the number of genes included, a preferably international database with a MLST scheme and already known alleles is required so that a sequence type (ST) can be assigned and isolates can be compared^8^. Such internationally accepted scheme and database on a whole genome scale is not yet available for all pathogens. For SNP-based WGS analysis, numerous software packages are available, but most, if not all, of these have no graphical interface and are run in a command-line environment, which is not feasible for the average routine laboratory. Retrospective WGS analysis of identified outbreaks may contribute to the development of adequate WGS data analysis pipelines for future outbreak detection^9^.

Here we report on the retrospective WGS analysis of two *S*. Enteritidis outbreaks that were taken as a case study to demonstrate the added value of WGS for outbreak investigation and to evaluate its feasibility for an average NRL or NRC. The two outbreaks were selected since for both the food and human isolates were available. Additionally, it concerned two geographically separated outbreaks, but occurring around the same time, of *S*. Enteritidis PT4 that were linked to non-commercial eggs from privately kept laying hens which were used to prepare desserts for social events. This would allow investigating the possibility of using WGS to distinguish between and within outbreaks or to confirm a common source of contamination. The WGS analysis was performed with user-friendly software and tools to demonstrate the feasibility of characterisation of outbreaks isolates by non-bioinformaticians, thereby facilitating its implementation in a routine NRL.

## Methods

**Epidemiological investigation**

In Belgium, the Federal Agency for the Safety of the Food Chain (FASFC) is responsible for sampling and investigation of food, while the Health Services of the Belgian Communities collect human samples. Epidemiological information such as age, symptoms, timeline and circumstances of the outbreaks were gathered by local health inspectors and by inspectors of the FASFC using a standard questionnaire. All collected information was transmitted to the Belgian National Reference Laboratory of Foodborne Outbreaks (NRL-FBO). For each of the two outbreaks, a case was defined as an individual who consumed a meal at the respective social event and who suffered from diarrhoea.

**Microbiological investigation**

The NRL-FBO received food samples, including leftovers, of both outbreaks for detection of *Salmonella*, which was performed according to ISO 6579:2002^10^. The Belgian National Reference Centre for *Salmonella* and *Shigella* (NRCSS) received *Salmonella* isolates from human cases of both outbreaks.

Isolates of both outbreaks were serotyped^11^ by the NRCSS and phage typed by Public Health England. MLVA^12^ was performed by the Belgian NRCSS.

The antimicrobial susceptibility of the *Salmonella* isolates was tested by determination of the minimal inhibitory concentration (MIC) of 14 antimicrobials in a Sensititre MIC plate EUVSEC with read-out on a Sensititre Vizion system. Following epidemiological cut-off values were applied: ampicillin 8 mg/l^13^, cefotaxime 0.5 mg/l^13^, ceftazidime 2 mg/l^13^, chloramphenicol 16 mg/l^13^, ciprofloxacin 0.064 mg/l^13^, colistin 2 mg/l^13^, gentamicin 2 mg/l^13^, meropenem 0.125 mg/l^13^, nalidixic acid 16 mg/l^13^, tetracycline 8 mg/l^13^, tigecycline 1 mg/l^14^ and trimethoprim 2 mg/l^13^. Epidemiological cut-off values were not available for azithromycin and sulphamethoxazole.

**Whole genome sequencing**

Genomic DNA of the outbreak isolates (Table 1) was extracted with the Qiagen Genomic-tip 100/G kit. The samples were sequenced at the EMBL GeneCore facility in 40-plex on a single lane of the Illumina HiSeq 2000 using 100 bp paired-end reads. FASTQ reads from all sequences were deposited at the WIV-ISP - *Salmonella* BioProject at NCBI (PRJNA289069).

**Table 1:** Overview of outbreak isolates selected for sequencing with the microbiological investigation results.

Outbreak	Isolate	Origin	Phage type	MLVA	Antimicrobial resistance
Flanders	S14FP01640	Chocolate mousse	PT4	3-10-5-4-1	Colistin
Flanders	S14FP01642	Chocolate mousse	PT4	3-10-5-4-1	Colistin
Flanders	S14BD01605	Human	PT4	3-10-5-4-1	Colistin
Flanders	S14BD01672	Human	PT4a	3-10-5-4-1	Colistin - ampicillin
Wallonia	S14FP01877	Raw egg (non-commercial)	PT4	3-10-5-4-1	Colistin
Wallonia	S14BD01753	Human	PT4	3-10-5-4-1	Colistin

**WGS data analysis**

All analyses were performed on a Windows 7 platform.

In CLC Genomics Workbench 8.0 the raw FASTQ reads were first trimmed to quality score limit 0.001 (Q30) with maximum 2 ambiguous nucleotides and reads with length below 15 nucleotides were discarded. These trimmed reads were then de novo assembled with automatic bubble and word size, in mapping mode ‘map reads back to contigs’ with scaffolding and a minimum contig length of 200 nucleotides.

On the server of the Center for Genomic Epidemiology^15^, the resulting contigs were uploaded to MLST 1.7^16^ with *Salmonella* *enterica* as MLST scheme, ResFinder 2.1^17^ and PlasmidFinder 1.2^18^. ResFinder was used to find all available antimicrobial resistance genes with minimum 98% identity and minimum 60% of their length. In PlasmidFinder, the database of Enterobacteriaceae was searched with an identity threshold of 95%. Additionally, raw FASTQ reads were uploaded to the CSI Phylogeny 1.0a^19^ server on which the SNP calling was run with *S*. Enteritidis P125109 (NC_011294) as reference genome, default input parameters as described by Kaas *et al.* 19 and a minimum Z-score of 1.96. The downloaded Newick file was used for visualisation of the phylogenetic tree in FigTree v1.4.2^20^. The downloaded vcf (variant call format) files were used for investigation of the position of the SNPs on the chromosome of *S*. Enteritidis P125109.

As PlasmidFinder results indicated that plasmids were present, trimmed reads were mapped to *S*. Enteritidis P125109 (NC_011294) in CLC Genomics Workbench 8.0 with default settings and unmapped reads were de novo assembled as described above. The resulting contigs were blasted to plasmids pSLA5 (NC_019002) and pSD107 (JX566770) and visualised as concentric rings with BLAST Ring Image Generator (BRIG)^21^.

## Results

The first outbreak occurred in Flanders at a social event with about 220 guests and where food was supplied by a catering service. The onset of the first symptoms was on April 23rd 2014. The outbreak extended to 45 cases with 5 hospitalised individuals and was reported to the Flemish Agency for Care and Health by pharmacists and general practitioners, who treated an unusually high number of people for diarrhoea. Since people who only ate dessert at the event also became ill, freshly prepared chocolate mousse and ice cream were indicated as possible cause of the outbreak. Due to shortage of commercial eggs, also non-commercial eggs from privately kept laying hens were used to prepare the chocolate mousse. Two samples from white and brown chocolate mousse were sent to the NRL-FBO and were tested positive for *Salmonella*. Raw eggs from privately kept laying hens, collected on April 24th and 25th, were also investigated, but these tested negative for *Salmonella*. The NRCSS received 11 *Salmonella* isolates from different human cases linked to this outbreak, which were isolated from stool in clinical laboratories. Two of these isolates were randomly selected for phage typing and WGS.

The second outbreak occurred in Wallonia on a social event which was attended by about 300 people and where a barbecue meal was prepared by volunteers. The onset of the first symptoms was on May 1st 2014. The number of cases was estimated at 40 and some people were hospitalised, but no number is available for the hospitalised cases. The hospital reported the outbreak to the Walloon-Brussels Health Inspection Service. The NRL-FBO received samples of mascarpone cheese, bacon, sausages, pork ribs and raw eggs (which were stored refrigerated). There were no left-overs of consumed tiramisu, which was prepared with the sampled mascarpone cheese and the sampled non-commercial eggs from privately kept laying hens. Chocolate mousse, prepared with commercial eggs, was also consumed, but no leftovers were available. The raw eggs from privately kept laying hens tested positive for *Salmonella*. One *Salmonella* isolate of a human case, which was isolated from stool in a clinical laboratory, was sent to the NRCSS.

All 15 outbreak isolates were serotyped as *S*. Enteritidis. Six collected food and human isolates related to the outbreak in Flanders and Wallonia (Table 1) were selected for sequencing and subtyping with available user-friendly software and tools. All 6 selected isolates (Table 1) were phage typed as PT4, except for isolate S14BD01672 which showed a PT4a phage type. MLVA resulted for all isolates in profile 3-10-5-4-1 (SENTR4-SENTR5-SENTR6-SENTR7-SE3).

The *de novo* assemblies consisted of 271 contigs on average (range 35-480) with an average N50 of 174624 (range 28773-405843). The MLST server^16^ typed all isolates as ST-11 (alleles: *aroc*-5/*dnan*-2/*hemd*-3/*hisd*-7/*pure*-6/*suca*-6/*thra*-11). No resistance genes were found by ResFinder^17^, with the exception of isolate S14BD01672, for which a perfect match to *bla*_TEM-1B_ (JF910132) was detected. These *in silico* results were phenotypically confirmed by the antimicrobial susceptibility tests, as for all outbreak isolates only a single resistance to colistin was observed, except for isolate S14BD01672 which had an additional resistance to ampicillin. As colistin resistance is often linked to mutations^22^, this will not be recognised by ResFinder, which only identifies resistance genes.

Results of PlasmidFinder^18^ pointed to the presence of plasmid pSLA5 in all 6 outbreak isolates and additionally to pSD107 in isolate S14BD01672. BRIG analyses are shown in Fig. 1 and 2. Absence of the region *srgB*-SELA5_RS23145-SELA5_p0022 on the pSLA5 plasmid of the outbreak isolates is most likely an artefact, because this region is also present on the chromosome of *S*. Enteritidis, so that reads mapping to this region on the chromosome are absent in the *de novo* assemblies of unmapped reads used for the BRIG analysis. This is also indicated by the coverage of this region in the read mapping to *S*. Enteritidis P125109, which is about 2 to 4 times higher than the average coverage of the read mapping to the chromosome of P125109.

**BRIG analysis pSLA5:**
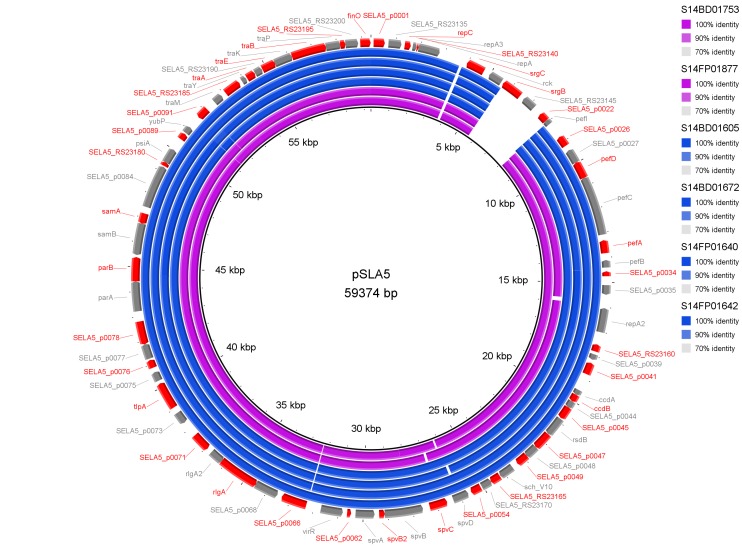
*De novo* assemblies of reads of the outbreak isolates that did not map to *S*. Enteritidis P125109 are shown as concentric rings with plasmid pSLA5 as reference on the inner black circle. Absence of colour in a ring indicates absence of the region. Isolates of the outbreak in Flanders are represented by a blue colour, those of the Walloon outbreak by a purple colour.

**BRIG analysis pSD107:**
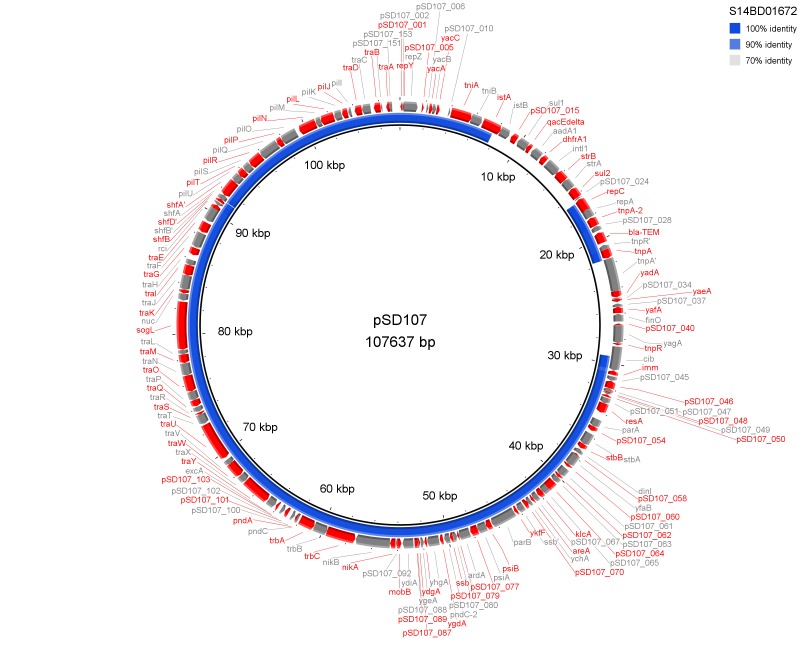
The *de novo* assembly of *S*. Enteritidis P125109 unmapped reads of outbreak isolate S14BD01672 is shown as a concentric ring with plasmid pSD107 as reference on the inner black circle. Absence of colour in the ring indicates absence of the region.

The analysis with CSI Phylogeny^19^ showed that there was a pairwise distance of 0 to 2 SNPs within the Flemish outbreak and more specifically, no SNPs between the food isolates (S14FP01640 and S14FP01642), 2 SNPs between the human isolates (S14BD01605 and S14BD01672) and 1 SNP between each of the food and each of the human isolates. Within the Walloon outbreak, no SNPs were observed between the food (S14FP01877) and human isolate (S14BD01753). Fifty-one to 53 SNPs were detected between the two outbreaks, which were distributed around the chromosome of *S*. Enteritidis P125109. Forty-five to 47 and 44 SNPs were observed between the reference P125109 and outbreak isolates of, respectively Flanders and Wallonia. A phylogenetic tree is presented in Fig. 3, clearly linking the human and food isolates within each outbreak and distinguishing between both outbreaks.

**CSI Phylogeny analysis:**
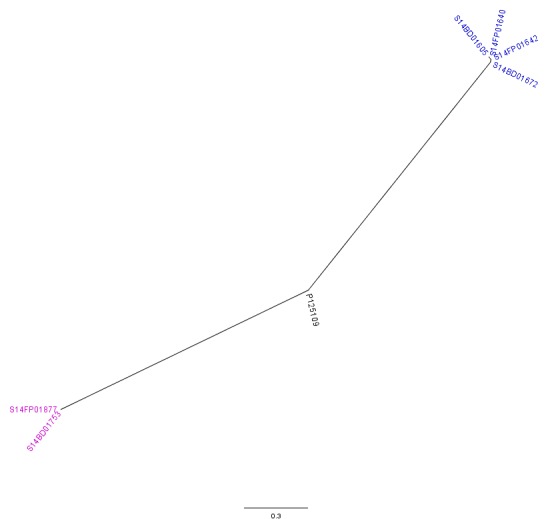
Radial phylogenetic tree of the 6 outbreak isolates with *S*. Enteritidis P125109 as reference. Isolates of the outbreak in Flanders are represented by a blue colour, those of the Walloon outbreak by a purple colour.

## Discussion

*S*. Enteritidis remains linked to egg-related outbreaks, albeit in the described outbreaks to non-commercial eggs from privately owned laying hens. In response to two geographically separated outbreaks occurring in the same time period in Belgium, the NRL-FBO received food samples from which *Salmonella* was isolated and the NRCSS received *Salmonella* isolates from human cases of these outbreaks. With the traditional epidemiological and microbiological investigations, *i.e.* phage typing and MLVA, the isolates of both outbreaks were classified as PT4 and variant PT4a, and as profile 3-10-5-4-1. Phage type PT4 and MLVA profile 3-10-5-4-1 are frequently observed for human isolates in Belgium. Therefore, the gold standard subtyping methods might not have been sufficiently discriminative to establish or exclude a common source of contamination for both outbreaks, which now could only be distinguished by their separate geographical location. However, there could still be a common origin, *e.g.* a common breeding flock of the laying hens bought by the private persons.

As WGS has been postulated as a universal subtyping method with ultimate resolution, the two outbreaks were retrospectively examined with WGS. Moreover, they were a good case study to investigate the feasibility of using WGS for outbreak investigation by a National Reference Laboratory, as both human and food isolates were available. An additional aspect that was evaluated in this study concerns the WGS data analysis tools, *i.e.* to see whether these are not restricted to expert bioinformaticians which are often not available in an average routine laboratory.

Therefore, the WGS data were analysed with user-friendly, albeit commercial, software (CLC Genomics Workbench) and the tools publicly available on the server of the Center for Genomic Epidemiology^15^, which not only allows for SNP analysis^19^, but also, amongst others, to explore the resistome (ResFinder^17^), to search for plasmids (PlasmidFinder^18^) and to assign a classical MLST^16^ sequence type based on 7 housekeeping genes. As mentioned above, an internationally accepted scheme and database on a whole genome scale for the allele-based data analysis workflow is not yet available for *Salmonella*, although commercially driven development efforts are ongoing, so that this type of analysis could not be performed in this study. Once the cgMLST for *S*. Enteritidis will be available, it would be interesting to reanalyse our dataset and to compare the results to those obtained with the SNP-based analysis. This will allow evaluating the impact of the selected type of data analysis workflow on the efficiency and accuracy of the outbreak investigation.

Similar to the traditional outbreak investigations, SNP analysis of the WGS data confirmed the association of food and human isolates in both outbreaks thereby proving the link between the contaminated eggs and the human cases who consumed these eggs. Moreover, and this in contrast to the gold standard subtyping methods, the SNP analysis was sufficiently discriminative to reveal a clear difference between the two outbreaks, *i.e.* the food isolates of the two outbreaks were not closely related. This clearly illustrates the utility of WGS and SNP analysis for a first indication of the source in the investigation of outbreaks. In our study, we observed about 52 SNPs difference between the two outbreaks, while only 0 to 2 SNPs difference within each outbreak. However, as previously suggested by Ashton *et al.* 9, it is difficult, if not impossible to set a single diversity threshold within a certain *Salmonella* outbreak, as it would depend on the size of the population that caused the outbreak, and hence it depends on the size of the facilities at the origin of the outbreak. As no sampling was done at the original sources of the outbreaks, namely the laying hens and their environment, or even at the distributor of these laying hens, the genetic diversity of the source population in the outbreaks described in this study could not be investigated. This sampling at the source, and an epidemiological investigation of this source, may be important for future outbreak investigations with WGS as the diversity of the source population may give an indication of the expected genetic diversity within outbreak isolates^9^. As such, more studies are still needed to contribute to the validation of SNP detection pipelines for this purpose.

The described analysis also shows that examination of mobile elements as plasmids can be useful for fine-tuning the results of a SNP analysis. One human outbreak isolate had a deviating phage type PT4a, which may be explained by presence of an additional plasmid carrying an antibiotic resistance gene (E. de Pinna, personal communication). Indeed, this mobile element also harbours a *bla*_TEM_ gene, which explains the ampicillin resistance observed phenotypically only in this isolate. As this mobile element was found in a human isolate, a possible hypothesis is that it might have been acquired during the foodborne infection. This could be further studied by analysis of multiple isolates from the same human case, which would also be interesting to examine the possible microevolution of a strain within a host.

The complete WGS analysis in this case study was performed on a Windows platform with currently available user-friendly software and tools which proves that WGS data analysis is not strictly restricted to bioinformaticians. As the use of WGS for characterisation of pathogens will only increase in the future, studies like these that are creating benchmarking datasets, may lead, in collaboration with hard core bioinformaticians, to the further development of user-friendly pipelines. This would imply that routine laboratories will no longer be solely dependent on bioinformaticians for WGS analyses and that WGS could be applied in real-time for diagnosis and outbreak investigations, if the infrastructure to generate the data in a short period of time is accessible to the routine laboratory.

This case study clearly demonstrated the added value of WGS as a complementary subtyping method during outbreak investigation, for isolates belonging to common circulating subtypes. The fact that the data analysis was done with user-friendly tools illustrates the feasibility of this technology for an average Reference Laboratory or Centre where bioinformatics expertise might be scarce. With the decreasing sequencing costs, WGS might become a replacing subtyping method, also in these environments. In this context, implementation of WGS in the average Reference Laboratories and Centres as routine characterisation method for *Salmonella* and other pathogens for surveillance and outbreak detection and investigation will benefit of sequencing of more outbreak and background isolates to create a database of circulating strains so that the diversity in the background population can be estimated and new outbreak isolates can be better discriminated. As outbreaks are not stopped by country borders, a European and/or international collaboration to set up such real-time WGS database would certainly be invaluable for future *Salmonella* outbreak detection and investigation.

## Competing Interests

The authors have declared that no competing interests exist.
